# Variants in a *cis*-regulatory element of *TBX1* in conotruncal heart defect patients impair GATA6-mediated transactivation

**DOI:** 10.1186/s13023-021-01981-4

**Published:** 2021-07-31

**Authors:** Xuechao Jiang, Tingting Li, Sijie Liu, Qihua Fu, Fen Li, Sun Chen, Kun Sun, Rang Xu, Yuejuan Xu

**Affiliations:** 1 Scientific Research Center, Xinhua Hospital, Affiliated to Shanghai Jiao Tong University School of Medicine, Shanghai, 200092 China; 2 Department of Pediatric Cardiology, Xinhua Hospital, Affiliated to Shanghai Jiao Tong University School of Medicine, Shanghai, 200092 China; 3 Medical Laboratory, Shanghai Children’s Medical Center, Affiliated to Shanghai Jiao Tong University School of Medicine , Shanghai, 200127 China; 4 Department of Pediatric Cardiology, Shanghai Children’s Medical Center, Affiliated to Shanghai Jiao Tong University School of Medicine, Shanghai, 200127 China

**Keywords:** TBX1, GATA6, *Cis*-regulatory element, Transcription, Pharyngeal arches, Conotruncal defect, Variant

## Abstract

**Background:**

*TBX1* (T-box transcription factor 1) is a major candidate gene that likely contributes to the etiology of velo-cardio-facial syndrome/DiGeorge syndrome (VCFS/DGS). Although the haploinsufficiency of TBX1 in both mice and humans results in congenital cardiac malformations, little has been elucidated about its upstream regulation. We aimed to explore the transcriptional regulation and dysregulation of *TBX1*.

**Methods:**

Different *TBX1* promoter reporters were constructed. Luciferase assays and electrophoretic mobility shift assays (EMSAs) were used to identify a *cis*-regulatory element within the *TBX1* promoter region and its *trans*-acting factor. The expression of proteins was identified by immunohistochemistry and immunofluorescence. Variants in the *cis*-regulatory element were screened in conotruncal defect (CTD) patients. In vitro functional assays were performed to show the effects of the variants found in CTD patients on the transactivation of *TBX1*.

**Results:**

We identified a *cis*-regulatory element within intron 1 of *TBX1* that was found to be responsive to GATA6 (GATA binding protein 6), a transcription factor crucial for cardiogenesis. The expression patterns of GATA6 and TBX1 overlapped in the pharyngeal arches of human embryos. Transfection experiments and EMSA indicated that GATA6 could activate the transcription of *TBX1* by directly binding with its GATA *cis*-regulatory element in vitro. Furthermore, sequencing analyses of 195 sporadic CTD patients without the 22q11.2 deletion or duplication identified 3 variants (NC_000022.11:g.19756832C > G, NC_000022.11:g.19756845C > T, and NC_000022.11:g. 19756902G > T) in the non-coding *cis*-regulatory element of *TBX1*. Luciferase assays showed that all 3 variants led to reduced transcription of *TBX1* when incubated with GATA6.

**Conclusions:**

Our findings showed that *TBX1* might be a direct transcriptional target of GATA6, and variants in the non-coding *cis*-regulatory element of *TBX1* disrupted GATA6-mediated transactivation.

**Supplementary Information:**

The online version contains supplementary material available at 10.1186/s13023-021-01981-4.

## Background

*TBX1*, encoding a T-box transcription factor, is located within the 1.5 Mb deletion of chromosome 22q11.2 [1–3]. *TBX1* is considered to be a major candidate gene of 22q11.2 deletion syndrome (22q11.2DS) [1–5], and point mutations or indels of *TBX1* were also identified in individuals with 22q11.2DS-like phenotypes [6–11]. Tbx1 is expressed in the pharyngeal arches and in the cardiac progenitors of the second heart field (SHF) [12–14], which gives rise to the outflow tract (OFT), right ventricle, and right and left atria [15–20]. Both the loss-of-function (LOF) and gain-of-function (GOF) of Tbx1 in mice results in congenital heart malformations, such as tetralogy of Fallot (TOF), double outlet right ventricle (DORV), and ventricular septal defect (VSD) [1, 4, 21–23].

Most research has focused on the TBX1 downstream transcription factors in cardiovascular development, such as *Isl1, Tbx5, Pax9, Fgf8, Fgf10, Pitx2 and Mef2c* [13, 14, 24–29]. Genetic studies indicate that TBX1 is a dosage-dependent transcriptional regulator [30, 31], but the mechanisms of *TBX1* gene upstream regulation remain largely unexplored. Previous studies have shown that TBX1 is regulated by sonic hedgehog (Shh) signaling [32, 33], vascular endothelial growth factor (VEGF) [34] and Ripply3 [35] or has a feedback loop with retinoic acid (RA) signaling [36], which are involved in pharyngeal arch development. However, how these signals affect the transcriptional regulation of *TBX1* remains unclear. Only Forkhead proteins have been reported to regulate the transcription of *Tbx1* directly [27, 33, 37], concerning that *Tbx1* is regulated by Shh signaling cascade through a Fox-binding site upstream of *Tbx1* in the pharyngeal endoderm [33]. *Cis*-regulatory elements such as enhancers and promoters are crucial for regulating gene expression [38, 39], and cardiac *cis*-regulatory elements functions are activated by a large number of both commonly expressed and cardiac-specific transcription factors, such as SNAI3, NKX2-5, GATA4, GATA6, SOX9, and NFATC1 [40]. Genome-wide association studies (GWAS) suggest that genetic variants associated with the risk of cardiovascular disease are enriched within annotated candidate *cis*-regulatory elements (cCREs) [41, 42]). The study of the *cis*-regulatory elements of *TBX1* will help to further understand the role of *TBX1* in the occurrence of CTD.

In this study, we found that GATA6 could positively activate *TBX1* transcription by directly binding to the *cis*-regulatory element within the *TBX1* promoter region, which contains GATA binding sites. We demonstrated that the expression patterns of TBX1 and GATA6 overlapped in the pharyngeal arches of human embryos. In addition, we identified 3 rare *TBX1* mutations within the *cis*-regulatory element in sporadic CTD patients without mutations in the coding region of a known CTD pathogenic gene and 22q11.2 deletions. Luciferase assays showed that these variants impaired the GATA6-mediated transcriptional activation of *TBX1*. These results provide novel insight into the molecular mechanisms associated with *TBX1* during the pathogenesis of CTD.

## Results

### Identification of a novel *cis*-regulatory element in human *TBX1* adjacent to the 5′ flanking region

**Fig. 1:**
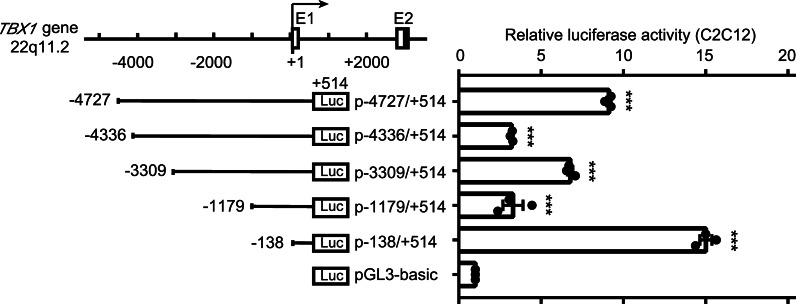
Deletion analysis identifies a 0.65-kb region around the *TBX1* TSS essential for transcriptional activity in the C2C12 cell line. (*Upper left*), genomic organization of the 5′ flanking region of human *TBX1*. The boxes show exons: blank box, non-coding exon; dark box, coding exon; E1, exon 1; E2, exon 2. Numbering shows the position relative to the TSS of the *TBX1* gene at g.19756703 (+1) of NC_000022.11. (*Bottom left*), schematic representation of 5′ truncated luciferase constructs. (*Right*), deletion analysis of the 5′ human *TBX1* flanking region. Relative luciferase activity of different 5′-serially deleted *TBX1* reporter constructs transfected in C2C12 cells, which were normalized to Renilla and represented as the fold increase when compared to the pGL3-basic vector. Data are shown as mean ± SEM, statistical significance was calculated by one-way ANOVA with Dunnett’s post hoc test, n = 3 independent experiments, ***p < 0.001 vs pGL3-basic

To identify potential *cis*-regulatory elements controlling *TBX1* transcription, a series of truncated human *TBX1* 5′-flanking regions were cloned into luciferase reporter vectors (Fig. 1). The subsequent dual-luciferase reporter analysis of these fragments showed that p-138/+ 514, which is located on chromosome 22: 19756565–19757216, significantly increased transcriptional activity by nearly 15-fold compared with the control vector in the C2C12 cell line (Fig. 1); and similar results were obtained in the NIH/3T3 cell line (Additional file 1: Fig. S1). This suggests that the *TBX1*-138/+ 514 region around the *TBX1* transcription start site (TSS) may be a *cis*-regulatory element, resulting in increased reporter expression.

We performed predictive analysis of cCREs around the *TBX1* TSS (Additional file 1: Fig. S2) with SCREEN (http://screen.encodeproject.org) [38], which is a web interface for searching and visualizing the registry of cCREs derived from ENCODE (Encyclopedia of DNA Elements) data (https://www.encodeproject.org/) [38, 43]. There is a cCRE (ENCODE Accession: EH38E2149905) possessing high DNase signals and high H3K4me3 signals, which is annotated as a proximal promoter-like signature (PLS). This cCRE-PLS genome browser was obtained from the UCSC Genome Bioinformatics Site (http://genome.cse.ucsc.edu/) [44], located on chromosome 22: 19756591–19756937 (Additional file 1: Fig. S2), and the *TBX1*-112/+ 235 region relative to the TSS, which is contained in the *TBX1*-138/+ 514 region.

### GATA6 significantly improved the promoter activity of *TBX1* by binding with a *cis*-regulatory element within the TBX1 promoter

**Fig. 2:**
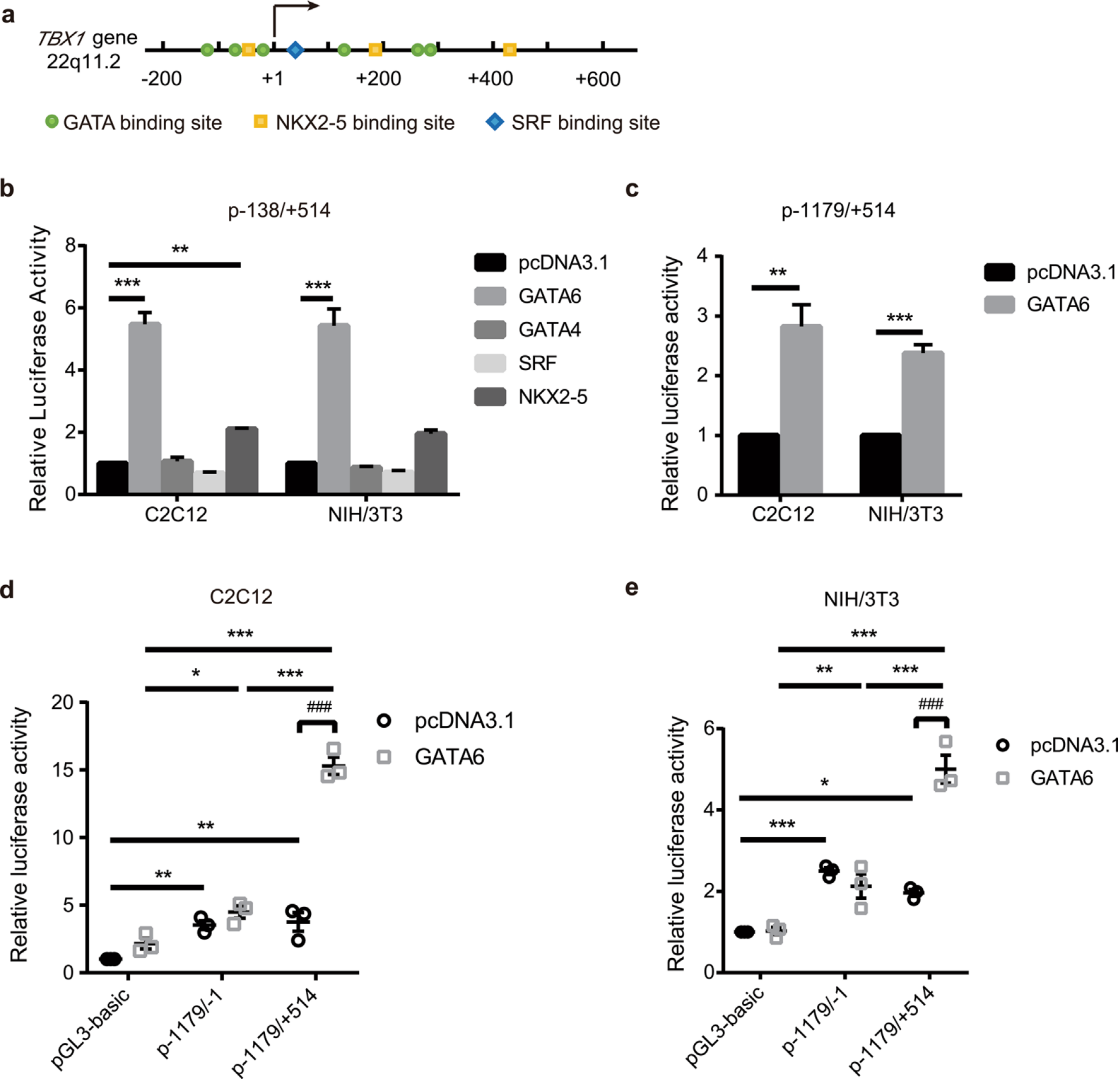
GATA6 transcriptional regulation of *TBX1* promoter. **a** Diagrammatic representation of the *TBX1 cis*-regulatory element showing putative potential GATA, NKX2-5, and SRF binding sites. **b** The influence of transcription factors on the p-138/+ 514 reporter gene in the C2C12 and NIH/3T3 cell lines. Luciferase analysis after cotransfection with luciferase reporter constructs containing the *TBX1 cis*-regulatory element *TBX1*-138/+ 514 and the expression vector of GATA6, GATA4, NKX2-5, SRF or empty vector (pcDNA3.1). The results were normalized to Renilla and presented as the fold induction over the pcDNA3.1 vector. Data are shown as mean ± SEM, significance was calculated by one-way ANOVA with Dunnett’s multiple comparisons test, n = 3 independent experiments, **p < 0.01 vs pcDNA3.1, ***p < 0.001 vs pcDNA3.1 in each cell line. **c** The influence of GATA6 on the *TBX1* promoter reporter gene p-1179/+ 514 in the C2C12 and NIH/3T3 cell lines. Luciferase analysis after cotransfection with luciferase reporter p-1179/+ 514 and GATA6 expression vector or empty vector (pcDNA3.1). The results were normalized to Renilla and presented as the fold induction over the pcDNA3.1 vector. Data are shown as mean ± SEM, two-tailed unpaired *t* test was used for statistical calculation, n = 3 independent experiments, **p < 0.01 vs pcDNA3.1, ***p < 0.001 vs pcDNA3.1 in each cell line. **d** and **e** The effects of the transcription factor GATA6 on the *TBX1* promoter reporter gene in C2C12 and NIH/3T3 cell lines. Luciferase analysis after cotransfection of luciferase reporter and GATA6 expression construct or empty vector (pcDNA3.1). The results were normalized to Renilla and presented as the fold induction over basal reporter (pGL3-basic + pcDNA3.1). Data are shown as mean ± SEM, n = 3 independent experiments. Significance was calculated by two-way ANOVA with Sidak’s post hoc test, ^###^*p* < 0.001. Significance was calculated by two-way ANOVA with Tukey's multiple comparisons test, *p < 0.05, **p < 0.01, ***p < 0.001

Cardiac *cis*-regulatory elements play important roles during transcriptional regulation by recruiting cardiac-specific transcription factors [40]. Therefore, to identify specific transcription factor binding sites within *TBX1*-138/+ 514, a non-exhaustive search for predicted candidate transcription factor binding sites was performed using JASPAR (http://jaspar.genereg.net/) [45], and the results showed that this region contained six GATA motifs, three NKX2-5 motifs and one serine response factor (SRF) motif (Fig. 2a). GATA4 and GATA6, two highly conserved transcription factors, are primarily expressed in the myocardium [46, 47], and are capable of binding to the same predicted GATA-binding sites [48–50]. We cotransfected the p-138/+ 514 reporter gene with individual expression vectors containing GATA4, GATA6, NKX2-5 or SRF in the C2C12 and NIH/3T3 cell lines (Fig. 2b). The results showed that GATA6 significantly increased luciferase activity by nearly sixfold compared with the control pcDNA3.1 vector, NKX2-5 slightly increased luciferase activity by only twofold, and GATA4 and SRF had no effects on luciferase activity (Fig. 2b).

Since *TBX1*-967/+ 185 [51] and *TBX1*-577/+ 439 [52] were used as promoters in previous literature, we used *TBX1*-1179/+ 514 as the *TBX1* promoter to test whether *TBX1*-1179/+ 514 could be activated by GATA6 as *TBX1*-138/+ 514. Similar results showing that GATA6 significantly increased *TBX1* promoter activity in the C2C12 and NIH/3T3 cell lines were acquired (Fig. 2c). Moreover, we compared the activation effects of GATA6 on *TBX1*-1179/-1 and *TBX1*-1179/+ 514. The GATA6 expression vector and the corresponding reporter genes p-1179/-1 or p-1179/+ 514 were cotransfected into the C2C12 (Fig. 2d) and NIH/3T3 cell lines (Fig. 2e). We found that the overexpression of GATA6 significantly increased the luciferase activity of p-1179/+ 514 compared with that of the control pcDNA3.1 vector but had almost no effect on p-1179/-1 (Fig. 2d, e), which further suggests that GATA6 regulates *TBX1* promoter activity by binding with the *TBX1* + 1/+ 514.

### Endogenous overlapping expression of GATA6 and TBX1 in the pharyngeal arches of human embryos

**Fig. 3:**
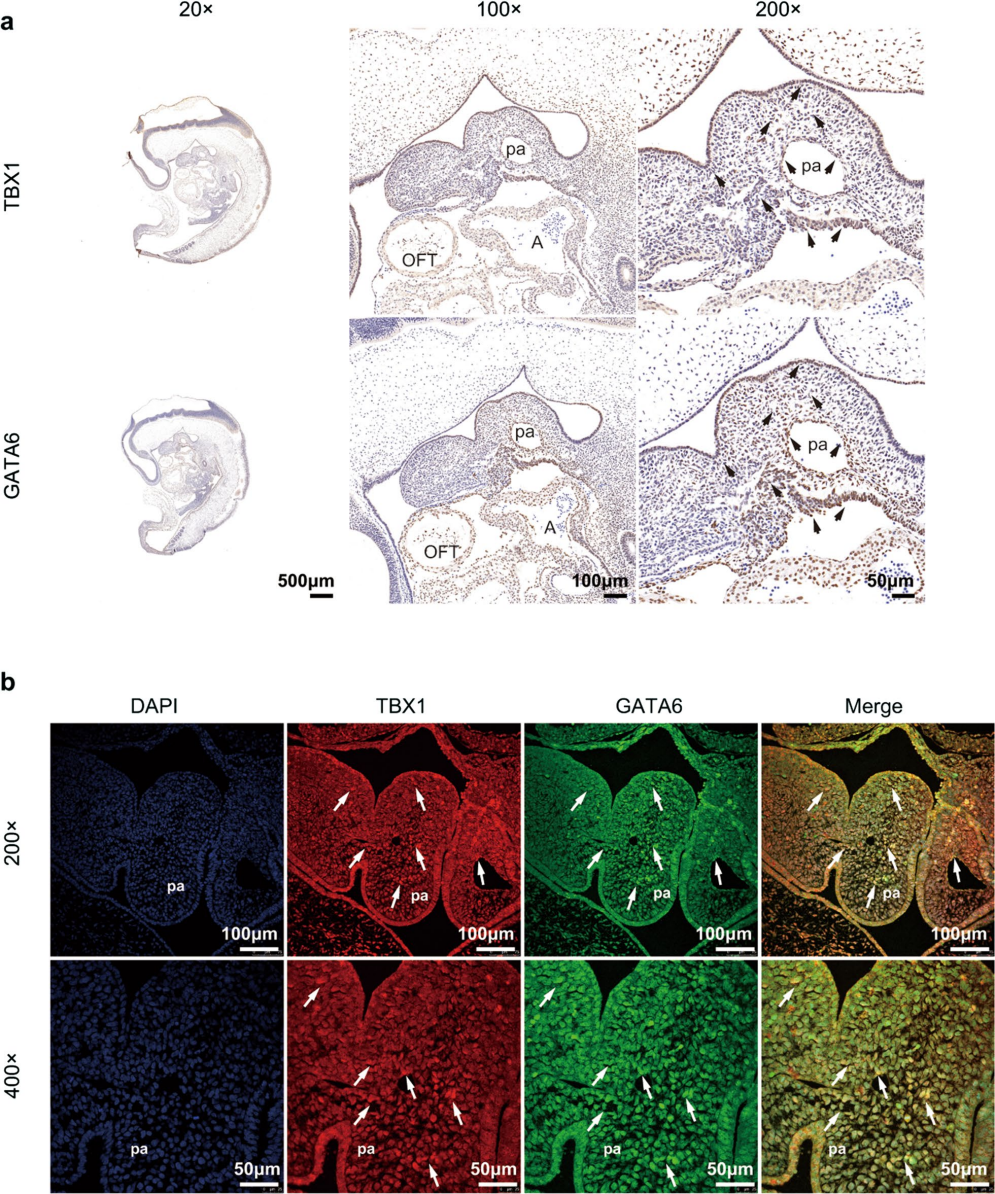
Histological staining analysis of TBX1 and GATA6 in human Carnegie Stage 13 (CS13) embryos. **a** Immunohistochemical staining for TBX1 and GATA6 in human CS13 embryos. Upper panel, TBX1 expression localized in pharyngeal arches (arrowheads); lower panel, GATA6 was expressed at pharyngeal arches (arrowheads) along with all atrial and ventricular chambers. Representative sagittal sections are shown (n = 3 embryos): dorsal is right; cranial is up. pa, pharyngeal arch; A, atrium; V, ventricle. Left panel, scale bars = 500 μm; middle panel, scale bars = 100 μm; right panel, scale bars = 50 μm. **b** Double immunofluorescence staining for TBX1 (red) and GATA6 (green) in human CS13 embryos. White arrows denote that both TBX1 and GATA6 are expressed in pharyngeal arches. Representative sagittal sections are shown (n = 3 embryos). pa, pharyngeal arch. Upper panel, scale bars = 100 μm; lower panel, scale bars = 50 μm

We performed immunohistochemistry (IHC) analyses for TBX1 and GATA6 in human Carnegie Stage 13 (CS13) embryos. As previously reported, TBX1 was predominantly expressed in the pharyngeal arches, whereas GATA6 was widely expressed in the pharyngeal arches, along with all atrial and ventricular chambers (Fig. 3a). To further determine the distinct but overlapping expression patterns of TBX1 and GATA6, we performed double-immunofluorescence staining. The examination of confocal projections revealed that TBX1 and GATA6 expression primarily overlapped in pharyngeal arches, which is a transient vertebrate-specific complex that participates in OFT development 27 (Fig. 3b).

### GATA6 directly binds with the *TBX1*-138/+514 *cis*-regulatory element within the *TBX1* promoter

**Fig. 4:**
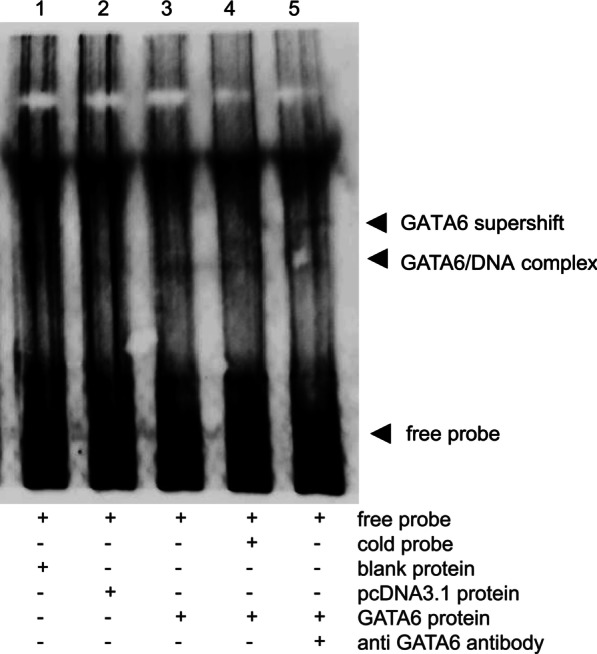
Electrophoretic gel mobility shift assays (EMSAs) revealed that GATA6 binds with the *cis*-regulatory element within the *TBX1* promoter directly. EMSAs were performed using biotin-labeled oligonucleotide probes containing + 115/+ 302 bp of *TBX1 cis*-regulatory element and in vitro-translated TNT blank protein, TNT pcDNA3.1 protein and TNT GATA6 protein by reticulocyte lysates, respectively. A protein-DNA complex was formed (lane 3), which could be inhibited by the addition of 120-fold molar excess unlabeled consensus GATA6 competitor DNA (lane 4) or antibody targeting GATA6 (lane 5). The arrows indicate unbound biotin-labeled free probe, GATA6-DNA complex and GATA6 antibody-GATA6-DNA supershift

To investigate whether GATA6 directly binds to the *TBX1*-138/+ 514 *cis*-regulatory element within the *TBX1* promoter, EMSA was performed using GATA6 protein and a biotin-labeled *TBX1* + 115/+ 302 probe containing three candidate GATA binding sites. An in vitro-translated GATA6 protein was verified by immunoblotting (Additional file 1: Fig. S3). The EMSA results showed that the GATA6 protein and the biotin-labeled probe *TBX1* + 115/+ 302 formed a protein-DNA supershift, which could be inhibited by the addition of a cold competition probe or an antibody targeting GATA6 (Fig. 4). Together, these results indicated that the human GATA6 protein was able to directly bind to the *TBX1*-138/+ 514 *cis*-regulatory element within the *TBX1* promoter.

### Variants within the TBX1 *cis*-regulatory element in CTD patients impair activation of the *TBX1* promoter by GATA6

**Fig. 5:**
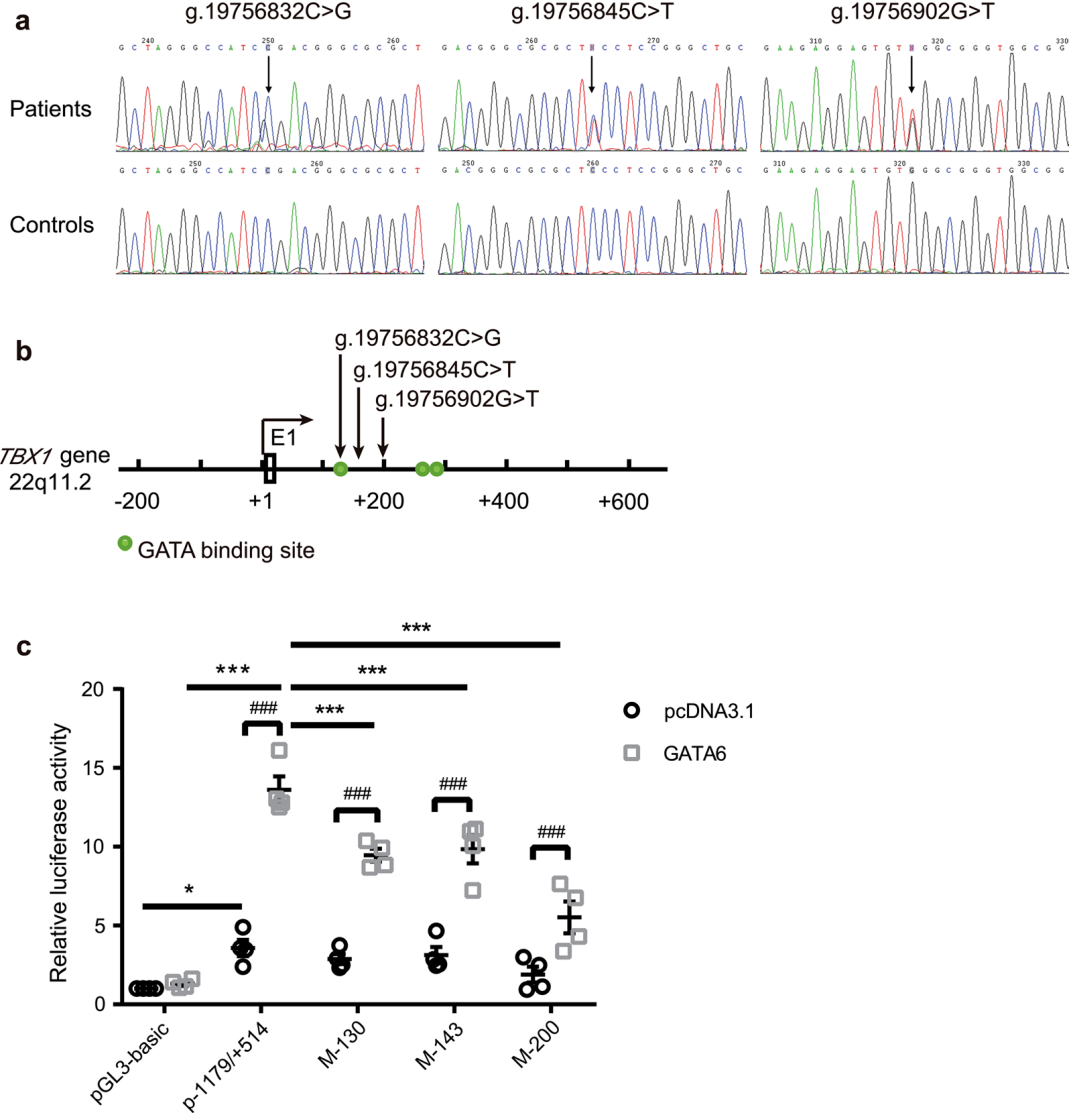
Mutation studies on the *cis*-regulatory element of the *TBX1* promoter associated with CTD. **a** Chromatograms of sequence variants within the *TBX1 cis*-regulatory element. (*Top*) shows the heterozygous mutation of patients, (*Bottom*) shows healthy controls, and all the variants are marked with arrows. **b** Schematic representation of the identified *TBX1 cis*-regulatory element variants in CTD patients. The transcription start site is at the position of g.19756703 (+1) in the first non-coding exon (E1). The numbers represent the genomic DNA sequences of the *TBX1* gene (NC_000022.11). **c** The effects of GATA6 on the *TBX1* mutation luciferase reporter genes M-130, M-143, and M-200. The WT or mutant *TBX1* promoter reporter gene were transfected alone into the C2C12 cell line or cotransfected with the GATA6 expression vectors. The luciferase activities were normalized to Renilla and presented as the fold increase relative to the activity of the reporter in the presence of an empty expression plasmid (pGL3-basic + pcDNA3.1). Patient variants are associated with a significant transcription activity reduction by GATA6. Data shown are mean ± SEM, n = 4 independent experiments. Significance was calculated by two-way ANOVA with Sidak’s post hoc test, ^###^*p* < 0.001. Significance was calculated by two-way ANOVA with Dunnett's multiple comparisons test, *p < 0.05 vs *p*-1179/+ 514, ***p < 0.001 vs p-1179/+ 514

In an effort to explore whether *TBX1 cis*-regulatory element variants are related to CTD, we screened the *TBX1*-138/+ 514 sequence in 195 patients with CTD. The proportions of CTD variations in these patients were as follows: 43.59% TOF, 23.08% DORV, 14.36% pulmonary atresia with ventricular septal defect (PA/VSD), 15.38% transposition of the great arteries (TGA), 2.56% interrupted aortic arch (IAA) and 1.03% persistent truncus arteriosus (PTA) (Table 1). None of the patients or control individuals presented copy number variants of 22q11.2 or any other chromosomal region (data not shown) [53]. We identified three heterozygous sequence variations in three patients (NC_000022.11:g.19756832C > G, NC_000022.11:g.19756845C > T and NC_000022.11:g. 19756902G > T), and all of the results were verified by Sanger sequencing (Fig. 5a). The clinical information and *TBX1*-138/+ 514 sequence mutations in patients with CTD are shown in Table 2. These three patients had PA, TOF and TGA respectively, combined with other congenital heart diseases (CHDs) such as patent ductus arteriosus (PDA), VSD and pulmonary stenosis (PS). The identified variant (NC_000022.11:g.19756832C > G) is located within the GATA binding site, whereas the other two variants (NC_000022.11:g.19756845C > T; NC_000022.11:g. 19756902G > T) are located between the GATA binding sites (Fig. 5b). None of the three variants were found in the 145 ethnically matched healthy controls, but two variants (NC_000022.11:g.19756845C > T and NC_000022.11:g. 19756902G > T) were reported in the 1000 Genomes database and gnomAD database (Table 2). Several known CHD pathogenic genes (*GATA4, GATA6, GATA5, HAND2, FOG2, NKX2-5, MEF2C, TBX1, TBX5, SOX7, PITX2,* and *MESP1*) were also screened in these three patients. There were no nonsynonymous variants identified in these known CHD pathogenic genes, including *TBX1*.

**Table 1:** Frequency and spectrum of CTD patients

Cardiac defects	Number	Percentage
TOF	85	43.59
DORV	45	23.08
PA/VSD	28	14.36
TGA	30	15.38
IAA	5	2.56
PTA	2	1.03
Total	195	100

TOF, tetralogy of Fallot; DORV, double outlet right ventricle; PA/VSD, pulmonary atresia with ventricular septal defect; TGA, transposition of the great arteries; IAA, interrupted aortic arch; PTA, persistent truncus arteriosus

**Table 2:** Clinical information and mutations of *TBX1 cis*-regulatory element in patients with CTD

Patient no	Gender	Age	Cardiac phenotype	Variant (NC_000022.11)	Location	Relative position*	1000 Genomes allele frequency	Gnomad allele frequency
91-PF	F	22 months	PA/VSD/PDA	g.19756832C > G	intron1	130 bp	–	–
193-PF	M	26 months	TOF	g.19756845C > T	intron 1	143 bp	0.0006000	0.00009866
40-PF	M	16 months	TGA/VSD/PS	g.19756902G > T	intron1	200 bp	0.0002000	0.00001317

F, female; M, male; PA, pulmonary atresia; VSD, ventricular septal defect; PDA, patent ductus arteriosus; TOF, tetralogy of Fallot; TGA, transposition of the great arteries; PS: pulmonary stenosis

*Variants were located relative to the transcription start site of *TBX1* gene at g.19756703 (+1) of NC_000022.11

We constructed vectors containing each mutation individually using the template *TBX1* promoter p-1179/+ 514, named M-130, M-143, and M-200. The wild-type (WT) or mutant *TBX1* promoter reporter gene were transfected alone into the C2C12 cell line or cotransfected with the GATA6 expression vectors to test whether these variants had any effects on *TBX1* transcriptional activation. The luciferase activity measurements showed that the mutation vector alone had no effect on *TBX1* promoter activity itself, but when transfected with GATA6, *TBX1* promoter activity decreased significantly compared with the WT (Fig. 5c). Taken together, these results revealed that these variants within the *TBX1 cis*-regulatory element inhibited GATA6-mediated transactivation of *TBX1*.

## Discussion

Our results identified a novel non-coding *cis*-regulatory element within the *TBX1* proximal promoter region, which is capable of binding with GATA6; and variants in the *cis*-element in CTD patients disrupt GATA6-mediated *TBX1* transactivation. CTD, the common type of cyanotic cardiac disease, is usually defined as malformations of the OFT, and *TBX1* participates in OFT development. The findings not only help to deepen the understanding of the molecular mechanism of *TBX1* transcription, but also provide ideas for the pathogenesis of CTD.

*TBX1*, the major candidate gene for 22q11.2DS, is a dosage-sensitive transcription factor contributing to the formation of the OFT during cardiogenesis [15, 31]. Understanding the mechanisms of *TBX1* upstream transcriptional regulation may uncover novel determinants of CTD. Transcriptional regulation is a significant mechanism that controls the spatial and temporal expression of genes during cardiac development, and non-coding *cis*-regulatory elements are critical participants in the transcriptional regulation process [54]. A series of truncated portions of the human *TBX1* flanking region were used to identify transcriptional *cis*-regulatory elements associated with *TBX1*. *TBX1*-138/+ 514, a 0.65 kb fragment around the *TBX1* TSS, was identified as a *cis*-regulatory element. As the *TBX1*-138/+ 514 region consists of a cCRE-PLS (ENCODE Accession: EH38E2149905, also named *TBX1*-112/+ 235), combined with previous reports [51, 52], this suggests that *TBX1*-138/+ 514 may be a *cis*-regulatory element within the *TBX1* promoter.

*Cis*-regulatory elements can alter transcriptional activity by recruiting DNA-binding transcription factors [40], and we identified potential transcription factor binding sites within the *TBX1*-138/+ 514 sequence, including GATA/NKX2-5/SRF motifs. Our results revealed a clear requirement for GATA6 to activate the *TBX1*-138/+ 514 *cis*-regulatory element within the *TBX1* promoter (*TBX1*-1179/+ 514). GATA6 belongs to the GATA family of zinc finger transcription factors that consists of six known family members (GATA1-6) [55]. GATA6 is capable of binding the GATA consensus motif (A/T)GATA(A/G) through zinc finger domains [48–50], which are essential *cis*-elements in the promoter or enhancer regions of a variety of cardiac-specific genes [49, 56]. Accumulating research underscores the important roles played by GATA binding motifs in the promoter regions of several myocardial genes, such as *wnt2* [57] and *Bmp-4* [48], which were both identified as direct downstream targets for Gata6. Previously, no evidence of a genetic interaction between GATA6 and *TBX1* has been reported. Using an EMSA, we showed that GATA6 could directly activate *TBX1* transcription by binding with the *cis*-regulatory element *TBX1*-138/+ 514. Our results also showed that TBX1 and GATA6, which are two key cardiac regulatory factors, showed an overlapping distribution in the pharyngeal arches of human embryos. Previous reports showed that *Semaphorin 3c* (*Sema3c*) transcript was downregulated in the OFT of *Tbx1*^−/−^ mouse embryos [17], and SEMA3C was regulated directly by GATA6 in the developing OFT [58, 59], suggesting that GATA6 may share a common molecular pathway with TBX1, or at least in part during OFT development [5, 59]. Our findings provide evidence of a molecular link between GATA6 and TBX1, confirming the above hypothesis. The conditional inactivation of Gata6 in cardiac neural crest cells results in failed OFT septation [58], and CHD patients with GATA6 mutations have OFT malformations [59–61], demonstrating a role for GATA6 in CTD.

TBX1 is critical for normal pharyngeal arch development, and both reductions and increases in TBX1 levels increase the risk of pharyngeal arch birth defects. Any allelic *TBX1* variations that cause TBX1 levels to fall below a threshold for proper pharyngeal arch development increase the risk of birth defects [34]. Although *TBX1* is the causative gene of 22q11.2, only a few mutations in the coding region of *TBX1* were identified in patients with the 22q11.2 syndrome phenotype without detectable deletion of 22q11.2, and it is possible that mutations in the *cis*-regulatory region of *TBX1* also play a role [9, 30, 37]. The identification of a novel *TBX1 cis*-regulatory element provided an opportunity to examine whether mutations within the *cis*-regulatory element occurred in patients with the 22q11DS phenotype but no chromosomal deletion. In our study, three mutations within the *TBX1 cis*-regulatory element region in CTD patients were consistently associated with significantly reduced transcriptional activation by GATA6. The mutation NC_000022.11:g.19756832C > G is located within the GATA binding site and is therefore expected to impact the GATA6-binding ability. The other two variants NC_000022.11:g.19756845C > T and NC_000022.11:g. 19756902G > T identified in this study were located adjacent to GATA binding sites, which may influence the biological function of the *TBX1* gene through other mechanisms. Therefore, further study is warranted. There are also reports of *TBX1 cis*-regulatory elements being involved in cardiac development [32, 33, 51, 62], such as a mutation of the Fox-binding site upstream of *Tbx1* abolishing Tbx1 expression in the pharyngeal endoderm [32, 33] and heterozygous variants NC_000022.11:g.19756055C > T and NC_000022.11: g.19756212A > C upstream of the *TBX1* TSS in VSD patients significantly decreasing the activity of the *TBX1* gene promoter [51]. In addition, studies have reported that variants in the regulatory region of other OFT development-related genes are also associated with CTD, such as *WNT5A* [63].

Although almost all 22q11.2DS patients are hemizygous for the deletion, highly variable genotypes and phenotypes are commonly observed [13, 64]; thus, it has been hypothesized that genes located outside the deleted region may serve as potential modifiers of 22q11.2DS [13, 65, 66]. Our findings uncover a novel mechanism of the GATA6 transcription factor in the regulation of the *TBX1* gene by a *cis*-regulatory element, and variants in the *cis*-regulatory element in CTD patients impair *TBX1* promoter activity by GATA6, thus providing a basis for the above hypothesis. In addition, with imaging in assessing the phenotype of CTD, computed tomography angiography (CTA) and contrast magnetic resonance angiography (MRA) precisely detect associated aortic, pulmonary venous and coronary anomalies, and other CHDs [67, 68], which will contribute to a better estimation of the variants and to a clear genotype–phenotype correlation.

## Conclusions

In conclusion, our data provide the first evidence of a genetic link between *TBX1* and GATA6 signaling. A GATA6-binding *cis*-regulatory element within the *TBX1* promoter was identified, and variants in the *cis*-regulatory element impaired GATA6-mediated *TBX1* transactivation. However, our study is preliminary. We do not have sufficient evidence to show that *TBX1* expression is directly dependent on the GATA6-binding *cis*-regulatory element during cardiovascular development in vivo, and the mutations in the *TBX1 cis*-regulatory element reported in this paper are causal for CTD. This is the limitation of our study. Further functional assays for the GATA6-TBX1 pathway in CTD patients need to be performed. Our study can be used as the first evidence for future research on the pathogenic non-coding variants in *cis*-regulatory elements in CTD. This is hoped to be further confirmed in other populations.

## Materials and methods

### Human samples

Human embryonic samples were obtained at Department of Obstetrics and Gynecology of Xinhua Hospital according to the procedure we previously reported [69]. In brief, the pregnant women who could not continue pregnancy for certain reasons, and elective medical termination of pregnancy were performed under the Maternity Protection Law of China. All the donors signed the informed consents from Xinhua Hospital, Affiliated to Shanghai Jiao Tong University School of Medicine. The study was approved by the Medical Ethics Committee of Xinhua Hospital (no. XHEC-C-2012-018). The aborted embryos were isolated with an Olympus stereomicroscope and categorized in accordance with the Carnegie stage (CS). The well-preserved specimens evaluated as normal, were fixed in 4% paraformaldehyde (PFA) overnight, embedded in paraffin sagitally, and then sectioned at the thickness of 4 μm.

Blood samples from 195 sporadic CTD patients (75 female, 120 male; median age 2.3 years) of Han ethnicity, which included 85 TOF, 45 DORV, 28 PA/VSD, 30 TGA, 5 IAA and 2 PTA patients, were collected in Shanghai Children’s Medical Center (SCMC) from November 2011 to January 2014 (Table 1) [53]. Clinical records like echocardiography, computed tomography angiocardiography and magnetic resonance angiocardiography [67, 68] were reviewed by pediatric cardiologists before recruitment, and patients who had extracardiac anomalies were excluded. None of which harbored 22q11.2 deletion or duplication tested by CNVplex [53]. A total of 145 healthy control individuals (57 female, 88 male, median age 6.43 years) were ethnically gender-matched and recruited from the same geographical area. DNA was extracted from the whole blood samples using the QIAamp DNA Blood Mini Kit (Qiagen, Duesseldorf, Germany). The human experimentations were approved by the Medical Ethics Committee of Xinhua Hospital (no. XHEC-C-2012-018) and Shanghai Children’s Medical Center (no. SCMC-201015). Fully written informed consent was obtained from all participants or their guardians. All procedures were in accordance with the ethical standards of the institutional and national research committees and the Declaration of Helsinki.

### Plasmid construction and site-directed mutagenesis

5′-nested polymerase chain reaction (PCR) primers (− 4727/+ 514 F, − 4336/+ 514 F, − 3309/+ 514 F, − 1179/+ 514 F, − 138/+ 514 F, − 1179/− 1 F, Additional file 2: Table S1) and 3′ PCR primers (common 3′ primer + 514 R, -1179/-1 R, Additional file 2: Table S1) were used to amplify serial deletion fragments of the 5′ end region of *TBX1* by the template *TBX1* (NC_000022.11). Numbering shows the position relative to the TSS of the *TBX1* gene at g.19756703 (+1) of NC_000022.11. Then, these fragments were subcloned into the *Kpn*I and *Xho*I sites of the pGL3-basic vector (Promega, Madison, Wisconsin, USA) with T4 DNA ligase to construct a series of truncated vectors such as p-4727/+ 514, p-4336/+ 514, p-3309/+ 514, p-1179/+ 514, p-138/+ 514, and p-1179/-1. All of the vectors were verified by Sanger sequencing and extracted by an Endo-free Plasmid Mini Kit (OMEGA, USA).

The p-1179/+ 514 vector was used as templates for all mutagenesis experiments. The mutant plasmids M-130, M-143 and M-200 were generated by site-directed mutagenesis using the QuikChange Site-Directed Mutagenesis Kit (Agilent Technologies, Santa Clara, CA, USA). The mutant primers are shown in Additional file 2: Table S1.

The wild-type GATA6 expression plasmid, pcDNA3.1(+)-GATA6 was kindly gifted by Professor Hiroyuki Yamagishi [59]. The GATA4, NKX2-5 and SRF wild-type plasmid was generated by subcloning the corresponding cDNA amplified from the vector pCMV6-Entry-GATA4/NKX2-5/SRF separately purchased from Origene (Rockville, USA) into the expression vector pcDNA3.1(+).

### Bioinformatics analysis of *TBX1*-138+514 *cis*-regulatory element

Predictive analysis of cCREs around the *TBX1* TSS were performed with the SCREEN (http://screen.encodeproject.org) [38, 43]. The cCRE-PLS genome browser were obtained from the UCSC Genome Bioinformatics Site (http:// genome.cse.ucsc.edu/) [44]. A non-exhaustive search for potential transcription factor binding sites of *TBX1*-138/+ 514 fragment using the JASPAR (http://jaspar.genereg.net/) [45].

### Cell culture and transfection

C2C12 (mouse myoblast) and NIH/3T3 (mouse embryo fibroblast) cells were obtained from Cell Bank of Chinese Academy of Sciences (Shanghai, China) and cultured at 37℃ in a 5% CO_2_ humidified incubator in DMEM (Gibco, Portland, OR, USA) supplemented with 10% fetal bovine serum (Gibco, Portland, OR, USA).

For luciferase reporter assay, cells were transfected with firefly luciferase reporter plasmid and Renilla luciferase expression plasmid pRL-TK (Promega, Madison, Wisconsin, USA) as an internal control per well using Fugene HD. The firefly and Renilla luciferase activities were measured 40 h after transfection by the Dual Luciferase reporter assay system (Promega, Madison, Wisconsin, USA). The transcriptional activities were represented as ratios of firefly luciferase activities over Renilla luciferase activities.

### Electrophoretic mobility shift assays (EMSA)

Plasmids pcDNA-3.1 and pCDNA3.1-GATA6 were used for in vitro protein synthesis with the TNT®T7 Quick Coupled transcription/translation system (Promega, Madison, Wisconsin, USA) according to the manufacturer’s instructions. The oligonucleotide probes (+ 115/+ 302 bp) acquired from the *TBX1 cis*-regulatory element were synthesized by PCR using 5′-end biotin-labeled primers (GATA-biotin + 115/+ 302, Additional file 2: Table S1). Unlabeled consensus oligonucleotides were used as competitors (GATA cons, Additional file 2: Table S1) [70]. The EMSA was performed using the LightShift Chemiluminescent EMSA kit (Thermo Scientific, USA) according to the manufacturers’ protocol. Briefly, for the binding reactions with volume 20 μl, 4uL TNT protein sample were incubated with 500 fmol of 5′-labeled biotin probes in the following buffer: 2 μl of 10 × binding buffer, 1 μl of 50% Glycerol, 1 μl of 1% NP-40, 1 μl of 1 M KCl, 1 μl of 100 mM MgCl2, 1 μl of 200 mM EDTA and 1 μl Salmon DNA. All reactions were incubated at room temperature for 20 min. For competitor lanes, a 120-fold excess of competitor oligonucleotides was added and incubated at room temperature for 10 min before the addition of biotin-labeled probe. For antibody lanes, assays were conducted in a similar manner with the following exceptions: addition of ∼5 μg of antibodies against GATA6 (sc-9055 X, Santa Cruz Biotechology Inc., USA) and incubation at room temperature for 20 min before the addition of the biotin-labeled DNA probe. The products were then separated by 6.5% nondenaturing polyacrylamide gels in 0.5 × TBE. The DNA–protein complex was electrotransferred to a positively charged of nylon membrane (Pierce), and the signal was detected by the chemiluminescent nucleic acid detection.

### Immunohistochemistry

Paraffin-embedded embryo sections underwent deparaffinization, hydration, and antigen retrieval with citrate buffer (Servicebio technology, China). Then quenched endogenous peroxidase activity with 3% H_2_O_2_, blocked in 3% BSA for 1 h at room temperature, followed by incubating with rabbit polyclonal antibody against TBX1 (Abcam Inc., ab18530, Cambridge, UK) or GATA6 (Santa Cruz Biotechnology Inc., sc-9055, USA) overnight at 4℃. Next, the secondary antibody, HRP-labelled anti-rabbit immunoglobulin (Gene Tech, Shanghai, China) was applied for 45 min at room temperature. Immunoreactions were visualized by diaminobenzidine substrate (DAB), and nuclei were stained with hematoxylin. Finally, sections were rinsed with water, dehydrated, cleared and mounted with neutral balsam. Immunohistochemical images were photographed with microscope slide scanner Pannoramic MIDI (3D HISTECH).

### Immunofluorescence

For immunofluorescence staining, the sections were deparaffinized, rehydrated, antigen repair with citrate buffer (Servicebio technology, China), washed in PBS briefly and blocked with 3% BSA for 1 h at room temperature. Primary antibodies rabbit anti-TBX1 (Abcam Inc., ab18530, Cambridge, UK), goat anti-GATA6 (Santa Cruz Biotechnology Inc., sc-7244, USA) diluted in 1% BSA and incubated at 4℃ overnight. Then sections were washed in 1 × PBS and stained with fluorescent secondary antibodies, Cy3-conjugated donkey anti-rabbit IgG (ServiceBio, GB21403, China) and FITC-conjugated donkey anti-goat IgG (ServiceBio, GB22404, China) for 50 min at room temperature, and nuclei were stained with 4′,6-diamidino-2-phenylindole (DAPI). Images were collected using Leica confocal microscope.

### Western blot analysis

Cells were lysed by RIPA buffer, mixed with SDS loading buffer, and denatured at 100 °C for 10 min. Proteins were separated by 10% SDS-PAGE gels (Beyotime Biotech Inc., Shanghai, China) and transferred electrophoretically onto polyvinylidene difluoride membrane (Merck Millipore, Darmstadt, Germany). Then the membranes were blocked with 5% skim milk for 2 h at room temperature, then incubated overnight at 4 °C with anti-GATA6 (Sigma-Aldrich, G2298) and anti-GAPDH (Sigma-Aldrich, G9545) antibodies. After the membranes were rinsed, they were incubated with the HRP-conjugated secondary antibody for 2 h at room temperature. Detection was performed by an enhanced chemiluminescence western blot detection system (Millipore, Billerica, MA, USA) according to the manufacturer’s instructions.

### Gene sequencing and mutation characterization

Mutation screening analysis of the *TBX1*-138/+ 514 *cis*-regulatory element sequence was performed in patients and control individuals by Sanger sequencing. The referential genomic DNA sequence of *TBX1* was derived from a nucleotide (NC_000022.11). Mutations in *TBX1*-138/+ 514 with a prevalence of under 0.1% in control populations (under 0.1% in both the 145 healthy controls and public variant databases, including 1000 Genomes and gnomAD) were regarded as candidate variants. The variants were validated by Sanger sequencing, and the primers used in the present study are summarized in Additional file 2: Table S1.

To validate the potential function of identified *TBX1*-138/+ 514 *cis*-regulatory element variants, the coding sequences and splicing sites of these known CHD pathogenic genes (*GATA4, GATA6, GATA5, HAND2, FOG2, NKX2-5, MEF2C, TBX1, TBX5, SOX7, PITX2, MESP1*) were also screened in CTD patients and controls using the EasyTarget® amplification kit (Genesky Biotechnologies Inc, Shanghai, China). Nonsynonymous mutations in the coding and splicing regions with a prevalence of under 0.1% in control populations (under 0.1% in both the 145 healthy controls and public variant databases, including 1000 Genomes, ExAC and gnomAD) were regarded as CHD pathogenic gene mutations.

### Statistical analysis

Data were analyzed by PRISM 6.0 (GraphPad Software Inc., La Jolla, CA, USA) software and were presented as the mean ± standard error of the mean (S.E.M.). The experiments were repeated at least three times independently. Two-tailed unpaired t-test was used to determine significant differences between two groups. One-way ANOVA with Dunnett’s post hoc test was used to analyze differences among three or more groups. Differences between two independent variables were analyzed by two-way ANOVA with Sidak’s multiple comparisons test, Tukey's multiple comparisons test or Dunnett’s multiple comparisons test. *P* < 0.05 were considered statistically significant.

## Supplementary Information

**Additional file 1. Fig. S1** Deletion analysis identifies a 0.65-kb region around the *TBX1* TSS essential for transcriptional activity in the NIH/3T3 cell line. **Fig. S2** UCSC Genome Browser views of cCREs around the *TBX1* TSS and the underlying DNase and ChIP data (hg38 human genomes). **Fig. S3** Verification of in vitro-translated GATA6 by reticulocyte lysates.

**Additional file 2. Table S1** Primers used for PCR, EMSA and Site-directed mutagenesis.

## Data Availability

The data sets used and/or analysed during the current study are available from the corresponding author on reasonable request.

## Abbreviations

TBX1: T-box transcription factor 1
VCFS: Velo-cardio-facial syndrome
DGS: DiGeorge syndrome
EMSAs: Electrophoretic mobility shift assays
CTD: Conotruncal defect
GATA6: GATA binding protein 6
22q11.2DS: 22Q11.2 deletion syndrome
SHF: Second heart field
OFT: Outflow tract
LOF: Loss-of-function
GOF: Gain-of-function
TOF: Tetralogy of Fallot
DORV: Double outlet right ventricle
VSD: Ventricular septal defect
Shh: Sonic hedgehog
VEGF: Vascular endothelial growth factor
RA: Retinoic acid
GWAS: Genome-wide association studies
cCREs: Candidate *cis*-regulatory elements
TSS: Transcription start site
ENCODE: Encyclopedia of DNA Elements
PLS: Promoter-like signature
SRF: Serine response factor
IHC: Immunohistochemistry
CS13: Carnegie stage 13
PA: Pulmonary atresia
TGA: Transposition of the great arteries
IAA: Interrupted aortic arch
PTA: Persistent truncus arteriosus
CHDs: Congenital heart diseases
PDA: Patent ductus arteriosus
PS: Pulmonary stenosis
WT: Wild-type
Sema3c: Semaphorin 3c
CTA: Computed tomography angiography
MRA: Magnetic resonance angiography
PFA: Paraformaldehyde
PCR: Polymerase chain reaction
DAB: Diaminobenzidine substrate
DAPI: 4′,6-Diamidino-2-phenylindole

## References

[1] Jerome LA, Papaioannou VE (2001). DiGeorge syndrome phenotype in mice mutant for the T-box gene, Tbx1. Nat Genet.

[2] Merscher S, Funke B, Epstein JA, Heyer J, Puech A, Lu MM, Xavier RJ, Demay MB, Russell RG, Factor S (2001). TBX1 is responsible for cardiovascular defects in velo-cardio-facial/DiGeorge syndrome. Cell.

[3] Zhao Y, Diacou A, Johnston HR, Musfee FI, McDonald-McGinn DM, McGinn D, Crowley TB, Repetto GM, Swillen A, Breckpot J (2020). Complete sequence of the 22q11.2 allele in 1053 subjects with 22q11.2 deletion syndrome reveals modifiers of conotruncal heart defects. Am J Human Genet.

[4] Lindsay EA, Vitelli F, Su H, Morishima M, Huynh T, Pramparo T, Jurecic V, Ogunrinu G, Sutherland HF, Scambler PJ (2001). Tbx1 haploinsufficieny in the DiGeorge syndrome region causes aortic arch defects in mice. Nature.

[5] Kodo K, Uchida K, Yamagishi H (2021). Genetic and cellular interaction during cardiovascular development implicated in congenital heart diseases. Front Cardiovasc Med.

[6] Pan Y, Wang ZG, Liu XY, Zhao H, Zhou N, Zheng GF, Qiu XB, Li RG, Yuan F, Shi HY (2015). A novel TBX1 loss-of-function mutation associated with congenital heart disease. Pediatr Cardiol.

[7] Rauch R, Hofbeck M, Zweier C, Koch A, Zink S, Trautmann U, Hoyer J, Kaulitz R, Singer H, Rauch A (2010). Comprehensive genotype-phenotype analysis in 230 patients with tetralogy of Fallot. J Med Genet.

[8] Zweier C, Sticht H, Aydin-Yaylagul I, Campbell CE, Rauch A (2007). Human TBX1 missense mutations cause gain of function resulting in the same phenotype as 22q11.2 deletions. Am J Human Genet.

[9] Yagi H, Furutani Y, Hamada H, Sasaki T, Asakawa S, Minoshima S, Ichida F, Joo K, Kimura M, Imamura S-I (2003). Role of TBX1 in human del22q112 syndrome. Lancet.

[10] Stoller JZ, Epstein JA (2005). Identification of a novel nuclear localization signal in Tbx1 that is deleted in DiGeorge syndrome patients harboring the 1223delC mutation. Hum Mol Genet.

[11] Paylor R, Glaser B, Mupo A, Ataliotis P, Spencer C, Sobotka A, Sparks C, Choi CH, Oghalai J, Curran S (2006). Tbx1 haploinsufficiency is linked to behavioral disorders in mice and humans: implications for 22q11 deletion syndrome. Proc Natl Acad Sci USA.

[12] Chieffo C, Garvey N, Gong W, Roe B, Zhang G, Silver L, Emanuel BS, Budarf ML (1997). Isolation and characterization of a gene from the DiGeorge chromosomal region homologous to the mouse Tbx1 gene. Genomics.

[13] Phillips HM, Stothard CA, Shaikh Qureshi WM, Kousa AI, Briones-Leon JA, Khasawneh RR, O'Loughlin C, Sanders R, Mazzotta S, Dodds R *et al*: Pax9 is required for cardiovascular development and interacts with Tbx1 in the pharyngeal endoderm to control 4th pharyngeal arch artery morphogenesis. Development. 2019;146(18). 10.1242/dev.177618 PMC6765178 31444215

[14] Ivins S, Lammerts van Beuren K, Roberts C, James C, Lindsay E, Baldini A, Ataliotis P, Scambler PJ (2005). Microarray analysis detects differentially expressed genes in the pharyngeal region of mice lacking Tbx1. Dev Biol.

[15] Chen L, Fulcoli FG, Tang S, Baldini A (2009). Tbx1 regulates proliferation and differentiation of multipotent heart progenitors. Circ Res.

[16] Francou A, Saint-Michel E, Mesbah K, Kelly RG (2014). TBX1 regulates epithelial polarity and dynamic basal filopodia in the second heart field. Development.

[17] Theveniau-Ruissy M, Dandonneau M, Mesbah K, Ghez O, Mattei MG, Miquerol L, Kelly RG (2008). The del22q11.2 candidate gene Tbx1 controls regional outflow tract identity and coronary artery patterning. Circ Res.

[18] Xu H, Morishima M, Wylie JN, Schwartz RJ, Bruneau BG, Lindsay EA, Baldini A (2004). Tbx1 has a dual role in the morphogenesis of the cardiac outflow tract. Development.

[19] Greulich F, Rudat C, Kispert A (2011). Mechanisms of T-box gene function in the developing heart. Cardiovasc Res.

[20] De Bono C, Thellier C, Bertrand N, Sturny R, Jullian E, Cortes C, Stefanovic S, Zaffran S, Theveniau-Ruissy M, Kelly RG (2018). T-box genes and retinoic acid signaling regulate the segregation of arterial and venous pole progenitor cells in the murine second heart field. Hum Mol Genet.

[21] Racedo SE, Hasten E, Lin M, Devakanmalai GS, Guo T, Ozbudak EM, Cai CL, Zheng D, Morrow BE (2017). Reduced dosage of beta-catenin provides significant rescue of cardiac outflow tract anomalies in a Tbx1 conditional null mouse model of 22q11.2 deletion syndrome. PLoS Genet.

[22] Hasten E, McDonald-McGinn DM, Crowley TB, Zackai E, Emanuel BS, Morrow BE, Racedo SE (2018). Dysregulation of TBX1 dosage in the anterior heart field results in congenital heart disease resembling the 22q11.2 duplication syndrome. Hum Mol Genet.

[23] Zhang Z, Huynh T, Baldini A (2006). Mesodermal expression of Tbx1 is necessary and sufficient for pharyngeal arch and cardiac outflow tract development. Development.

[24] Pane LS, Fulcoli FG, Cirino A, Altomonte A, Ferrentino R, Bilio M, Baldini A (2018). Tbx1 represses Mef2c gene expression and is correlated with histone 3 deacetylation of the anterior heart field enhancer. Dis Models Mech.

[25] Gao S, Moreno M, Eliason S, Cao H, Li X, Yu W, Bidlack FB, Margolis HC, Baldini A, Amendt BA (2015). TBX1 protein interactions and microRNA-96–5p regulation controls cell proliferation during craniofacial and dental development: implications for 22q11.2 deletion syndrome. Hum Mol Genet.

[26] Nowotschin S, Liao J, Gage PJ, Epstein JA, Campione M, Morrow BE (2006). Tbx1 affects asymmetric cardiac morphogenesis by regulating Pitx2 in the secondary heart field. Development.

[27] Hu T, Yamagishi H, Maeda J, McAnally J, Yamagishi C, Srivastava D (2004). Tbx1 regulates fibroblast growth factors in the anterior heart field through a reinforcing autoregulatory loop involving forkhead transcription factors. Development.

[28] Liao J, Aggarwal VS, Nowotschin S, Bondarev A, Lipner S, Morrow BE (2008). Identification of downstream genetic pathways of Tbx1 in the second heart field. Dev Biol.

[29] Aggarwal VS, Morrow BE (2008). Genetic modifiers of the physical malformations in velo-cardio-facial syndrome/DiGeorge syndrome. Dev Disabil Res Rev.

[30] Scambler PJ (2010). 22q11 deletion syndrome: a role for TBX1 in pharyngeal and cardiovascular development. Pediatr Cardiol.

[31] Zhang Z, Baldini A (2008). In vivo response to high-resolution variation of Tbx1 mRNA dosage. Hum Mol Genet.

[32] Garg V, Yamagishi C, Hu T, Kathiriya IS, Yamagishi H, Srivastava D (2001). Tbx1, a DiGeorge syndrome candidate gene, is regulated by sonic hedgehog during pharyngeal arch development. Dev Biol.

[33] Yamagishi H, Maeda J, Hu T, McAnally J, Conway SJ, Kume T, Meyers EN, Yamagishi C, Srivastava D (2003). Tbx1 is regulated by tissue-specific forkhead proteins through a common Sonic hedgehog-responsive enhancer. Genes Dev.

[34] Stalmans I, Lambrechts D, De Smet F, Jansen S, Wang J, Maity S, Kneer P, von der Ohe M, Swillen A, Maes C (2003). VEGF: a modifier of the del22q11 (DiGeorge) syndrome?. Nat Med.

[35] Okubo T, Kawamura A, Takahashi J, Yagi H, Morishima M, Matsuoka R, Takada S (2011). Ripply3, a Tbx1 repressor, is required for development of the pharyngeal apparatus and its derivatives in mice. Development.

[36] Yutzey KE (2010). DiGeorge syndrome, Tbx1, and retinoic acid signaling come full circle. Circ Res.

[37] Maeda J, Yamagishi H, McAnally J, Yamagishi C, Srivastava D (2006). Tbx1 is regulated by forkhead proteins in the secondary heart field. Dev Dyn Off Publ Am Assoc Anatomists.

[38] Moore JE, Purcaro MJ, Pratt HE, Epstein CB, Shoresh N, Adrian J, Kawli T, Davis CA, Dobin A, Consortium EP (2020). Expanded encyclopaedias of DNA elements in the human and mouse genomes. Nature.

[39] Hocker JD, Poirion OB, Zhu F, Buchanan J, Zhang K, Chiou J, Wang TM, Zhang Q, Hou X, Li YE, et al. Cardiac cell type-specific gene regulatory programs and disease risk association. Sci Adv. 2021;7(20). 10.1126/sciadv.abf1444 PMC8121433 33990324

[40] Lee D, Kapoor A, Safi A, Song L, Halushka MK, Crawford GE, Chakravarti A (2018). Human cardiac cis-regulatory elements, their cognate transcription factors, and regulatory DNA sequence variants. Genome Res.

[41] Maurano MT, Humbert R, Rynes E, Thurman RE, Haugen E, Wang H, Reynolds AP, Sandstrom R, Qu H, Brody J (2012). Systematic localization of common disease-associated variation in regulatory DNA. Science.

[42] Smemo S, Campos LC, Moskowitz IP, Krieger JE, Pereira AC, Nobrega MA (2012). Regulatory variation in a TBX5 enhancer leads to isolated congenital heart disease. Hum Mol Genet.

[43] Snyder MP, Gingeras TR, Moore JE, Weng Z, Gerstein MB, Ren B, Hardison RC, Stamatoyannopoulos JA, Graveley BR, Consortium EP (2020). Perspectives on ENCODE. Nature.

[44] Navarro Gonzalez J, Zweig AS, Speir ML, Schmelter D, Rosenbloom KR, Raney BJ, Powell CC, Nassar LR, Maulding ND, Lee CM (2021). The UCSC genome browser database: 2021 update. Nucleic Acids Res.

[45] Fornes O, Castro-Mondragon JA, Khan A, van der Lee R, Zhang X, Richmond PA, Modi BP, Correard S, Gheorghe M, Baranasic D (2020). JASPAR 2020: update of the open-access database of transcription factor binding profiles. Nucleic Acids Res.

[46] Stennard FA, Costa MW, Elliott DA, Rankin S, Haast SJP, Lai D, McDonald LPA, Niederreither K, Dolle P, Bruneau BG (2003). Cardiac T-box factor Tbx20 directly interacts with Nkx2-5, GATA4, and GATA5 in regulation of gene expression in the developing heart. Dev Biol.

[47] Charron F, Nemer M (1999). GATA transcription factors and cardiac development. Semin Cell Dev Biol.

[48] Nemer G, Nemer M (2003). Transcriptional activation of BMP-4 and regulation of mammalian organogenesis by GATA-4 and -6. Dev Biol.

[49] Pikkarainen S (2004). GATA transcription factors in the developing and adult heart. Cardiovasc Res.

[50] Morrisey EE, Ip HS, Tang Z, Parmacek MS (1997). GATA-4 activates transcription via two novel domains that are conserved within the GATA-4/5/6 subfamily. J Biol Chem.

[51] Wang H, Chen D, Ma L, Meng H, Liu Y, Xie W, Pang S, Yan B (2012). Genetic analysis of the TBX1 gene promoter in ventricular septal defects. Mol Cell Biochem.

[52] Jiang H, Li L, Yang H, Bai Y, Jiang H, Li Y (2014). Pax2 may play a role in kidney development by regulating the expression of TBX1. Mol Biol Rep.

[53] Zhang X, Xu Y, Liu D, Geng J, Chen S, Jiang Z, Fu Q, Sun K (2015). A modified multiplex ligation-dependent probe amplification method for the detection of 22q11.2 copy number variations in patients with congenital heart disease. BMC Genom.

[54] Zhang Z, Baldini A (2010). Manipulation of endogenous regulatory elements and transgenic analyses of the Tbx1 gene. Mammalian Genome Off J Int Mammalian Genome Soc.

[55] Lentjes MH, Niessen HE, Akiyama Y, de Bruine AP, Melotte V, van Engeland M (2016). The emerging role of GATA transcription factors in development and disease. Expert Rev Mol Med.

[56] Peterkin T, Gibson A, Loose M, Patient R (2005). The roles of GATA-4, -5 and -6 in vertebrate heart development. Semin Cell Dev Biol.

[57] Alexandrovich A, Arno M, Patient RK, Shah AM, Pizzey JA, Brewer AC (2006). Wnt2 is a direct downstream target of GATA6 during early cardiogenesis. Mech Dev.

[58] Lepore JJ, Mericko PA, Cheng L, Lu MM, Morrisey EE, Parmacek MS (2006). GATA-6 regulates semaphorin 3C and is required in cardiac neural crest for cardiovascular morphogenesis. J Clin Investig.

[59] Kodo K, Nishizawa T, Furutani M, Arai S, Yamamura E, Joo K, Takahashi T, Matsuoka R, Yamagishi H (2009). GATA6 mutations cause human cardiac outflow tract defects by disrupting semaphorin-plexin signaling. Proc Natl Acad Sci USA.

[60] Gharibeh L, Komati H, Bosse Y, Boodhwani M, Heydarpour M, Fortier M, Hassanzadeh R, Ngu J, Mathieu P, Body S (2018). GATA6 regulates aortic valve remodeling, and its haploinsufficiency leads to right-left type bicuspid aortic valve. Circulation.

[61] Sharma A, Wasson LK, Willcox JA, Morton SU, Gorham JM, DeLaughter DM, Neyazi M, Schmid M, Agarwal R, Jang MY, et al. GATA6 mutations in hiPSCs inform mechanisms for maldevelopment of the heart, pancreas, and diaphragm. *eLife.* 2020;, 9. 10.7554/eLife.53278 PMC7593088 33054971

[62] Wang E, Nie Y, Fan X, Zheng Z, Hu S (2019). Intronic polymorphisms in gene of second heart field as risk factors for human congenital heart disease in a chinese population. DNA Cell Biol.

[63] Li P, Li H, Zheng Y, Qiao B, Duan W, Huang L, Liu W, Wang H (2015). Variants in the regulatory region of WNT5A reduced risk of cardiac conotruncal malformations in the Chinese population. Sci Rep.

[64] Unolt M, Versacci P, Anaclerio S, Lambiase C, Calcagni G, Trezzi M, Carotti A, Crowley TB, Zackai EH, Goldmuntz E (2018). Congenital heart diseases and cardiovascular abnormalities in 22q11.2 deletion syndrome: from well-established knowledge to new frontiers. Am J Med Genet Part A.

[65] Guo T, McDonald-McGinn D, Blonska A, Shanske A, Bassett AS, Chow E, Bowser M, Sheridan M, Beemer F, Devriendt K (2011). Genotype and cardiovascular phenotype correlations with TBX1 in 1022 velo-cardio-facial/DiGeorge/22q11.2 deletion syndrome patients. Hum Mutation.

[66] Guris DL, Duester G, Papaioannou VE, Imamoto A (2006). Dose-dependent interaction of Tbx1 and Crkl and locally aberrant RA signaling in a model of del22q11 syndrome. Dev Cell.

[67] Abdel Razek AAK, Al-Marsafawy H, Elmansy M (2019). Imaging of pulmonary atresia with ventricular septal defect. J Comput Assist Tomogr.

[68] Razek AA, Saad E, Soliman N, Elatta HA (2010). Assessment of vascular disorders of the upper extremity with contrast-enhanced magnetic resonance angiography: pictorial review. Jpn J Radiol.

[69] Jiang X, Li T, Li B, Wei W, Li F, Chen S, Xu R, Sun K (2021). SOX7 suppresses endothelial-to-mesenchymal transitions by enhancing VE-cadherin expression during outflow tract development. Clin Sci.

[70] Mwinyi J, Hofmann Y, Pedersen RS, Nekvindova J, Cavaco I, Mkrtchian S, Ingelman-Sundberg M (2010). The transcription factor GATA-4 regulates cytochrome P4502C19 gene expression. Life Sci.

