# Supplementary material for: Variants in a cis-regulatory element of TBX1 in conotruncal heart defect patients impair GATA6-mediated transactivation
Source: Orphanet J Rare Dis. 2021 Jul 31;16:334. doi: 10.1186/s13023-021-01981-4 (PMC8325851; doi:10.1186/s13023-021-01981-4)
Supplement: Supplementary file 1 — Additional file 1. Fig. S1 Deletion analysis identifies a 0.65-kb region around the TBX1 TSS essential for transcriptional activity in the NIH/3T3 cell line. Fig. S2 UCSC Genome Browser views of cCREs around the TBX1 TSS and the underlying DNase and ChIP data (hg38 human genomes). Fig. S3 Verification of in vitro-translated GATA6 by reticulocyte lysates. [file 13023_2021_1981_MOESM1_ESM.docx]

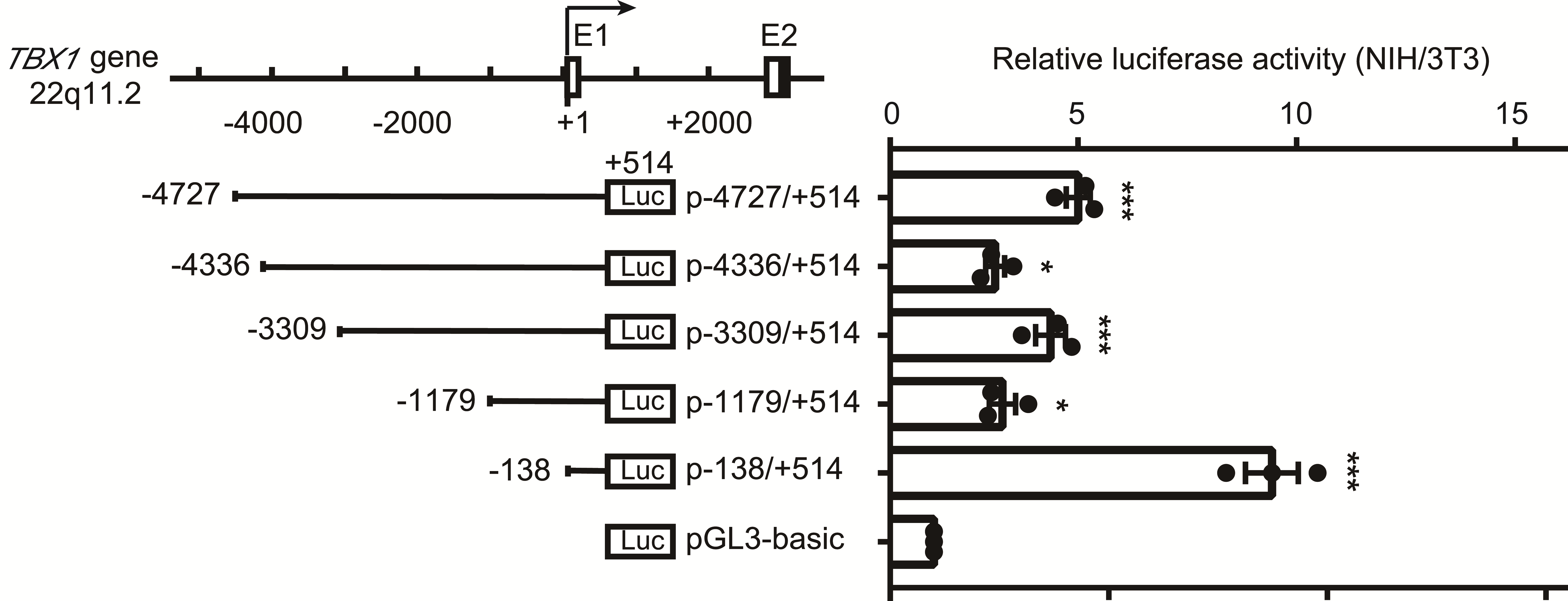


**Fig. S1** Deletion analysis identifies a 0.65-kb region around the *TBX1* TSS essential for transcriptional activity in the NIH/3T3 cell line. **(***Upper left*), genomic organization of the 5’ flanking region of human *TBX1*. The boxes show exons: blank box, non-coding exon; dark box, coding exon; E1, exon 1; E2, exon 2. Numbering shows the position relative to the TSS of *TBX1* gene at g.19756703 (+1) of NC_000022.11. (*Bottom left*), schematic representation of 5’ truncated luciferase constructs. (*Right*), deletion analysis of the 5’ human *TBX1* flanking region. Relative luciferase activity of different 5’-serially deleted *TBX1* reporter constructs transfected in NIH/3T3 cells, which were normalized to Renilla and represented as the fold increase when compared to the pGL3-basic vector. Data are shown as mean ± SEM, statistical significance was calculated by one-way ANOVA with Dunnett’s *post hoc* test, n=3 independent experiments, **p* < 0.05 vs pGL3-basic, ****p* < 0.001 vs pGL3-basic.


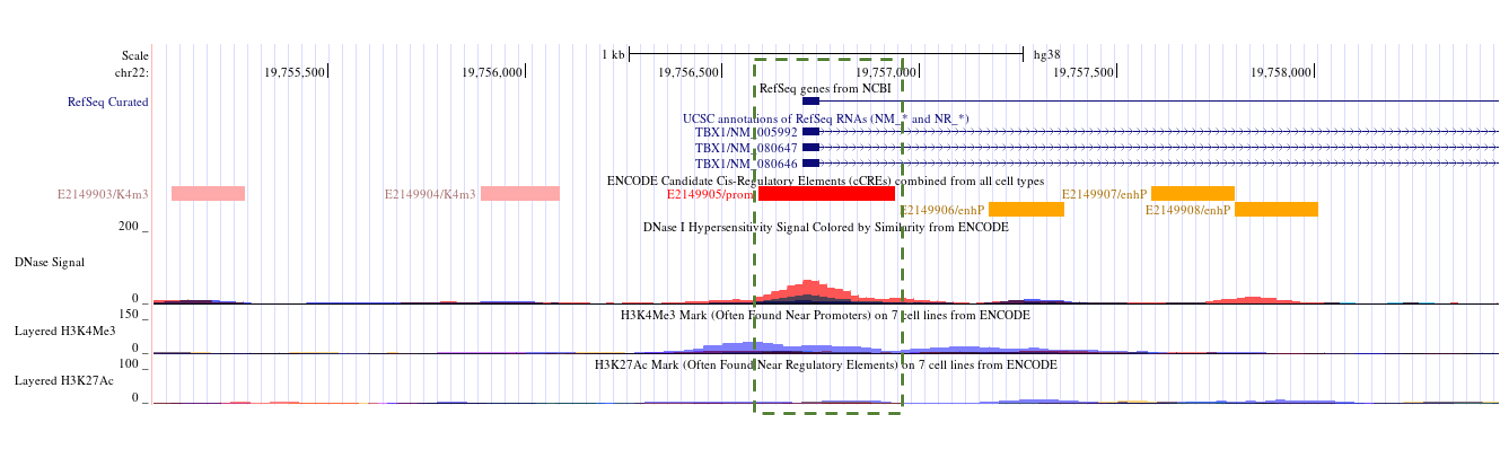


**Fig. S2** UCSC Genome Browser views of cCREs around the *TBX1* TSS and the underlying DNase and ChIP data (hg38 human genomes).The cCREs classification, DNase-H3K4me3 in pink, PLS (promoter-like signatures) in red, pELS (proximal enhancer-like signatures) in orange. The last three tracks show the DNase, H3K4me3 and H3K27ac signals. EH38E2149905 in green box is a cCRE-PLS that has high DNase, high H3K4me3, and low H3K27ac signals.


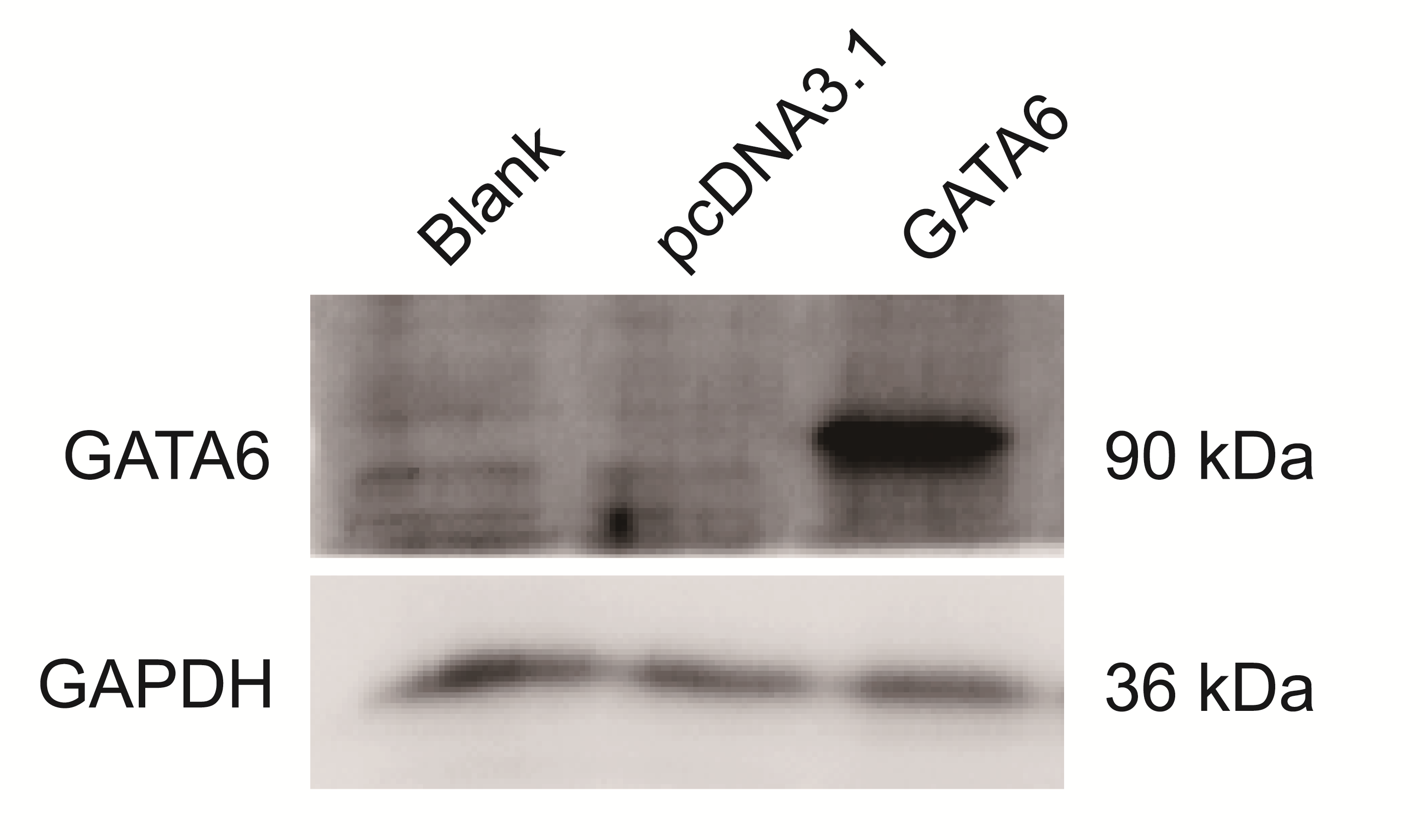


**Fig. S3** Verification of *in vitro*-translated GATA6 by reticulocyte lysates. Representative immunoblots of GATA6 with *in vitro*-translated TNT blank protein, TNT pcDNA3.1 protein and TNT GATA6 protein. GAPDH was used as internal control. Repeat 3 independent experiments.
