# Supplementary material for: Variants in a cis-regulatory element of TBX1 in conotruncal heart defect patients impair GATA6-mediated transactivation
Source: Orphanet J Rare Dis. 2021 Jul 31;16:334. doi: 10.1186/s13023-021-01981-4 (PMC8325851; doi:10.1186/s13023-021-01981-4)
Supplement: Supplementary file 2 — Additional file 2. Table S1 Primers used for PCR, EMSA and Site-directed mutagenesis. [file 13023_2021_1981_MOESM2_ESM.docx]

**Table S1** Primers used for PCR, EMSA and Site-directed mutagenesis

| Primer Name | | Sequence (5’-3’) | |
| --- | --- | --- | --- |
| PCR | | |  |
| -4727/+514 F | GGGGTACCTGGGAAGGCGGGAGACCAGACAGAGTT | | |
| -4336/+514 F  -3309/+514 F  -1179/+514 F  -138/+514 F  +514 R  -1179/-1 F  -1179/-1 R  EMSA | GGGGTACCAGGGAGCAGAGTGGACCGAGAG  GGGGTACCTATGTGCCCACACTCCCATGTATGT  GGGGTACCGTCAAGAGGTGGGAGTGGAGA  GGGGTACCGATGTCTCAGCCCAGGCCCTAGCCT  CCGCTCGAGACCAGAAGGGCGACGGGAAGA  GGGGTACCGTCAAGAGGTGGGAGTGGAGA  CCGCTCGAGCCGCACCTCCACACCCGCG | | |
| GATA-biotin+115/+302 F  GATA-biotin+115/+302 R | 5’-Biotin-CGGGCTAGGGCCATCCGAC  5’-Biotin-CACCGCCTCCATCGCGCAG | | |
| GATA cons F  GATA cons R  Site-directed mutagenesis | CACTTGATAACAGAAAGTGATAACTCTACG  CGTAGAGTTATCACTTTCTGTTATCAAGTG | | |
| M-130 F  M-130 R  M-143 F  M-143 R  M-200 F  M-200 R | GGCTAGGGCCATC**G**GACGGGCGCGCTC  GAGCGCGCCCGTC**C**GATGGCCCTAGCC  CGACGGGCGCGCT**T**CCTCCGGGCTGC  GCAGCCCGGAGG**A**AGCGCGCCCGTCG  GGTGAAGAGGAGTGT**T**GGCGGGTGGCGGGG  CCCCGCCACCCGCC**A**ACACTCCTCTTCACC | | |

F, forward primers; R, reverse primers. Underlines represent restriction sites placed in the primers, GGTACC: *Kpn*Ⅰ, CTCGAG: *Xho*Ⅰ. GATA cons comprises a representative GATA consensus sequence. Boldface indicates nucleotide changes in the oligo sequences.
